# Inhaled therapies targeting prostacyclin pathway in pulmonary hypertension due to COPD: systematic review

**DOI:** 10.3389/fmed.2023.1217156

**Published:** 2023-08-29

**Authors:** Abdullah A. Alqarni, Abdulelah M. Aldhahir, Heba M. Bintalib, Jaber S. Alqahtani, Rayan A. Siraj, Mansour Majrshi, Abdulkareem A. AlGarni, Abdallah Y. Naser, Sara A. Alghamdi, Hassan Alwafi

**Affiliations:** ^1^Department of Respiratory Therapy, Faculty of Medical Rehabilitation Sciences, King Abdulaziz University, Jeddah, Saudi Arabia; ^2^Respiratory Therapy Unit, King Abdulaziz University Hospital, Jeddah, Saudi Arabia; ^3^Respiratory Therapy Department, Faculty of Applied Medical Sciences, Jazan University, Jazan, Saudi Arabia; ^4^Department of Respiratory Care, King Saud bin Abdulaziz University for Health Sciences, Jeddah, Saudi Arabia; ^5^King Abdullah International Medical Research Centre, Jeddah, Saudi Arabia; ^6^Department of Respiratory Care, Prince Sultan Military College of Health Sciences, Dammam, Saudi Arabia; ^7^Department of Respiratory Care, College of Applied Medical Sciences, King Faisal University, Al Ahsa, Saudi Arabia; ^8^National Heart and Lung Institute, Imperial College London, London, United Kingdom; ^9^Respiratory Medicine, Royal Brompton Hospital, London, United Kingdom; ^10^King Abdulaziz Hospital, The Ministry of National Guard Health Affairs, Al Ahsa, Saudi Arabia; ^11^King Saud bin Abdulaziz University for Health Sciences, College of Applied Medical Sciences, Al Ahsa, Saudi Arabia; ^12^Department of Applied Pharmaceutical Sciences and Clinical Pharmacy, Faculty of Pharmacy, Isra University, Amman, Jordan; ^13^Respiratory Care Department, Mediclinic Almurjan Hospital, Jeddah, Saudi Arabia; ^14^Faculty of Medicine, Umm Al-Qura University, Mecca, Saudi Arabia

**Keywords:** pulmonary hypertension, COPD, prostacyclin, group 3 PH, treprostinil, iloprost, ventavis, tyvaso

## Abstract

**Background:**

Pulmonary hypertension due to chronic obstructive pulmonary disease (COPD) and interstitial lung disease (ILD) is classified as group 3 pulmonary hypertension. Inhaled treprostinil, a prostaglandin I_2_ analogue also known as prostacyclin, has recently been approved as a first drug for patients with pulmonary hypertension secondary to ILD. However, due to a lack of evidence, no therapies are currently approved for those with COPD-associated pulmonary hypertension. Thus, this systematic review aims to summarise the current evidence to assess the impact of inhaled prostaglandin I_2_ analogue use on the pulmonary hemodynamics, exercise function, lung function, and gas exchange in patients with pulmonary hypertension due to COPD.

**Methods:**

We systematically searched the electronic databases of Medline, Embase, Scopus and Cochrane from inception to 1 February 2023. Studies of adult patients with a confirmed diagnosis of COPD-associated pulmonary hypertension who received inhaled drugs targeting the prostacyclin pathway were included in the systematic review. Case reports, systematic reviews, conference abstracts with no full text, non-full-text articles, non-English manuscripts and book chapters were excluded from this systematic review. A risk-of-bias assessment was carried out for the studies included in this review, using two different Cochrane risk-of-bias tools for randomised and non-randomised clinical trials.

**Results:**

A total of four studies met our inclusion criteria and were included in this systematic review. The results of one prospective clinical trial showed an improvement in the pulmonary hemodynamics (e.g., cardiac index, cardiac output and mean pulmonary artery pressure) in response to inhaled prostacyclin use in patients with pulmonary hypertension secondary to COPD. However, the severity of dyspnoea, lung function, exercise capacity and gas exchange were not affected when inhaled prostacyclin was used for patients with COPD-related pulmonary hypertension.

**Conclusion:**

This systematic review demonstrated that although inhaled prostacyclin does not seem to improve COPD-related outcomes (e.g., lung function and exercise capacity), short-term use of inhaled prostacyclin has the potential to reduce mean pulmonary artery pressure and pulmonary vascular resistance without impairing ventilation-perfusion mismatch. Further studies with larger sample sizes are warranted.

**Systematic review registration:**

CRD42022372803, https://www.crd.york.ac.uk/prospero/display_record.php?RecordID=372803.

## 1. Introduction

Pulmonary hypertension is defined as an increased mean pulmonary artery pressure and is associated with increased mortality and morbidity (1). Pulmonary hypertension has been subdivided into five groups based on the underlying cause, clinical presentation and treatment strategies (2). Pulmonary hypertension due to lung diseases and hypoxia is classified as group 3. Among lung diseases, chronic obstructive pulmonary disease (COPD) is one of the most common lung diseases associated with the development of pulmonary hypertension (1).

Pulmonary hypertension is a common complication of COPD. The prevalence of COPD-associated pulmonary hypertension varies between 20.5 and 90.8% (3–11). More importantly, the presence of pulmonary hypertension in patients with COPD has been reported to be associated with further impairment in lung function and reduction in exercise capacity and quality of life (12) and worse clinical outcomes particularly in those with severe pulmonary hypertension (13). The underlying aetiology of COPD-associated pulmonary hypertension remains unclear; however, different pathways (prostaglandin I_2_, nitic oxide and endothelin) are thought to be involved. Among these pathways, we have previously shown that altered prostanoids (including prostaglandin I_2_) pathways may play a pivotal role in pulmonary artery remodelling in cigarette smoke-induced COPD (14, 15), suggesting that prostanoids pathways may serve as a potential therapeutic target for pulmonary hypertension due to COPD.

Prostaglandin I_2_ is one of the major metabolites of arachidonic acid (AA). AA is produced by the hydrolysis of cellular phospholipids *via* the action of phospholipase A_2_ (PLA_2_) and is converted first to unstable prostaglandin H_2_ (PGH_2_) by cyclooxygenase activity, and then to different prostanoids, including prostaglandin I_2_. Prostaglandin I_2_ activates the prostacyclin (IP) receptor, which leads to the relaxation of pulmonary vascular smooth muscle and inhibits platelet activation (16). The reduction of endogenous prostaglandin I_2_ represents the rationale for targeting the prostaglandin I_2_ pathway for the treatment of pulmonary hypertension (17). Compensating for the loss of prostaglandin I_2_ by using a prostacyclin analogue or prostacyclin receptor agonist has been shown to improve exercise capacity, symptoms, and the haemodynamic index in patients with group 1 pulmonary hypertension (18–23). Although drugs targeting this pathway are used as a therapeutic target for patients with group 1 pulmonary arterial hypertension, they are currently not approved for patients with pulmonary hypertension due to COPD.

Results from COMPERA study demonstrated that patients with severe pulmonary hypertension in COPD may benefit from oral administration of prostaglandin I_2_ analogue and other approved therapies for use in patients with group 1 pulmonary hypertension (endothelin receptor antagonists and phosphodiesterase type 5 inhibitors) (24). Despite the fact that pulmonary hypertension due to COPD may be different from pulmonary hypertension due to ILD in terms of clinical phenotype, treatment response and outcomes (13), drugs targeting inhaled prostaglandin I_2_ analogue have shown promising results in patients with group 3 pulmonary hypertension, particularly those with interstitial lung disease (ILD) −associated pulmonary hypertension. Recently, inhaled treprostinil, a prostaglandin I_2_ analogue, was approved following a randomised clinical trial that showed improvement in exercise capacity in those with pulmonary hypertension secondary to ILD (group 3). Given that the potential benefit of inhaled prostaglandin I_2_ analogue use in patients with pulmonary hypertension due to COPD has not been systemically reviewed before, we propose here the first systematic review that aims to summarise the current evidence to assess the impact of inhaled prostaglandin I_2_ analogue use on the pulmonary hemodynamics, the severity of dyspnoea, exercise capacity, lung function, and gas exchange in patients with pulmonary hypertension due to COPD.

## 2. Materials and methods

The systematic review protocol was prospectively registered on PROSPERO (registration number: CRD42022372803). Studies retrieved were sent to EndNote and then entered into Rayyan software,^1^ where blinding of the investigators was achieved. AAlq and HB evaluated the titles and abstracts of all studies against the inclusion criteria, and any disagreements were resolved by a third reviewer (AAld). If the title and abstract were not informative enough, reviewers read the entire manuscript to determine whether the study should be included. In addition, we checked the references for further sources. We extracted the data following the Preferred Reporting Items for Systematic Reviews and Meta-Analyses (PRISMA) guidelines and MOOSE guidelines for systematic review and meta-analysis of observational studies (25, 26). A standardised data extraction sheet was used to extract data from suitable full-text articles. The extracted data are summarised and presented in Table 1.

**Table 1:** Summary of included studies.

Authors, years of publication and country	Study design	COPD severity: *n* (male)	Type of inhaled PGI_2_ analogue, (dose in μg, duration, groups)	Diagnostic test to confirm PH, (mean/median mPAP)	Baseline PVR, CO, and CI	Outcome	Findings
United States (27)	Prospective, multi-center, open-label pilot study	GOLD 2: 9 (5)	Inhaled Treprostinil Dose: N.A Duration: up to 16 weeks Only one group: outcomes were assessed at baseline and after 16 weeks	PH is confirmed with RHC, median mPAP is 46 mmHg	PVR median (range): 729 (211–1,491) dynes/s/cm^−5^ CO median (range): 3.9 (2.2–7.6) L/min CIx median (range): 2.4 (1.3–4.0) L/min/m^2^	Primary outcome Gas exchange: PaO_2_, PaCO_2_, SaO_2_, A-a gradient Secondary outcomes World Health Organization functional class (WHO-FC) Dyspnoea: the modified Borg scale Exercise capacity: 6MWT Lung function: TLC, FEV_1_, FEV_1_%, FVC, FEV_1_/FVC%, RV/TLC, DLCO Quality of life: (SGRQ)	Gas exchange: there were no significant difference between baseline and week 16 in PaO_2_, PaCO_2_, SaO_2_, A-a gradient WHO- FC: there was no significant difference between baseline and week 16. Modified Borg dyspnoea scores: there was no significant difference in symptoms between baseline and week 16. Exercise capacity: there was no significant difference in 6MWT between baseline and week 16 PFT: there were significant differences between baseline and week 16 in FEV_1_ (L) (Median 1.50 L vs. 1.32 L; *p* = 0.004) and FVC (L) (Median, 2.73 L vs. 2.46 L; *p* = 0.027) QOL: there were no significant differences in SGRQ total scores between baseline and week.
Switzerland (28)	Prospective, randomised, double- blind, single center, cross-over trial	GOLD 2: 16 (10)	Inhaled Iloprost Dose: 10 and 20 μg Duration: N.A Group 1: placebo (normal saline) Group 2: 10 μg Group 3: 20 μg	PH is confirmed with RHC, mean mPAP is 31.3 mmHg	PVR mean (SD): 266.5 (123.5) dynes/s/cm^−5^ CO: N.A CIx mean (SD): 5.3 (1.3) L/min/m^2^	Primary outcome Exercise capacity: 6MWT Secondary outcomes Gas exchange: VO_2_ and VCO_2_ Dyspnoea: the modified Borg scale	Exercise capacity: there was no significant difference in mean ± SD in 6MWT between all groups. Gas exchange: there were significant differences in Peak oxygen consumption (VO_2 peak_) during the 6MWT over the three study groups (*p* = 0.002). The iloprost 10 ug group: VO_2 peak_ as compared to placebo (estimated difference of the means: −76 mL/min, 95% CI: −122−−31 mL/min, *p* = 0.002) and VCO_2exercise_ (EDOM: −70 mL/min, 95% CI: −115−−26 mL/min, *p* = 0.004). There were significant differences across all study groups in SpO_2_ at rest Group 10 ug (EDOM: −1.0, 95% CI: −1.9−−0.1%, *p* = 0.035). Group 20 ug (EDOM: −2.2, 95% CI: −3.1 − 1.2%, *p* < 0.001). Oxygen saturation after exercise was significantly declined in iloprost 20 ug group (EDOM: −2.4, 95% CI: −3.4–0.0%; *p* = 0.047) as compared to placebo. Modified Borg dyspnoea scores: there was no significant difference between all groups.
China (29)	Prospective, single-center, open-label trial	GOLD 3: 67 (40)	Inhaled Iloprost Dose: 20 μg Duration: 10 min Only one group: Outcomes were assessed at baseline and post treatment.	PH is confirmed with RHC, mean mPAP is 63.1 mmHg	PVR mean (SD): 491.9 (244.5) dynes/s/cm^−5^ CO mean (SD): 5.2 (1.7) L/min CIx mean (SD): 3.3 (0.9) L/min/m^2^	Primary outcome Hemodynamic index: PVR, PAWP, mPAP, RAP, CIx, CO and MAP. Secondary outcomes Gas exchange: PaO2, PaCO_2_, SaO_2_, D_A-_a O_2_ and Qs/Qt.	Hemodynamic index There was a significant difference between baseline and after Iloprost in PVR (baseline mean ± SD: 491.9 ± 244 dyn·s·cm^−5^ vs. after Iloprost mean ± SD: 429.6 ± 243 dyn·s·cm^−5^; *p* < 0.01). There was a significant difference between baseline and after Iloprost in mPAP (baseline mean ± SD: 39.6 ± 10.4 mmHg vs. after Iloprost mean ± SD: 37.4 ± 10.9 mmHg; *p* < 0.01). There was a significant difference between baseline and after Iloprost in cardiac index (baseline mean ± SD: 3.3 ± 0.9 L·min^−1^·m^−2^ vs. after Iloprost mean ± SD: 3.5 ± 0.9 L·min^−1^·m^−2^; *p* < 0.01). There was a significant difference between baseline and after Iloprost in cardiac output (baseline mean ± SD: 5.2 ± 1.7 L·min^−1^ vs. after Iloprost mean ± SD: 5.6 ± 1.9 L·min^−1^ *p* < 0.01). Gas exchange: There were no significant differences between baseline and after Iloprost in PaO2, PaCO_2_, SaO_2_, D_A_-O_2_ and Qs/Qt.
United States (30)	Prospective, single-cente (a single-day study)	GOLD 3: 10 (10)	Inhaled Iloprost Dose: 2.5 μg Duration: 30 min and 2 h Only one group: Outcomes were assessed at baseline and post treatment at 30 min and 2 h	PH is confirmed with echocardiography, mean mPAP is 40.6 mmHg	PVR: N.A CO: N.A CI: N.A	Primary outcome Gas exchange: D_A–a_ O_2_, (VE/VO_2_), (VE/VCO_2_) Secondary outcome Exercise capacity: 6MWT Lung function: spirometry and diffusion capacity	Gas exchange D_A–a_ O_2_ Single dose of Iloprost: There was a significant difference between baseline and after 30 min of Iloprost use (baseline mean ± SD: 30.7 ± 7.7 mmHg vs. after Iloprost mean ± SD: 27.0 ± 6.8 mmHg; *p* < 0.01). Second dose of Iloprost: There was a significant difference between baseline and after 30 min of Iloprost use (baseline mean ± SD: 30.7 ± 7.7 mmHg vs. after Iloprost mean ± SD: 26.9 ± 7.0 mmHg; *p* < 0.01). After 2 h. of treatment discontinuation: There was no significant difference between baseline and after 2 h. of Iloprost use. VE/VO_2_ Single dose of Iloprost: There was a significant difference between baseline and after 30 min of Iloprost use (baseline mean ± SD: 75.4 ± 21.0 vs. after Iloprost mean ± SD: 59.1 ± 13.1; *p* < 0.05). Second dose of Iloprost: There was a significant difference between baseline and after 30 min of Iloprost use (baseline mean ± SD: 75.4 ± 21.0 vs. after Iloprost mean ± SD: 57.7 ± 13.7; *p* < 0.05). After 2 h. of treatment discontinuation: There was no significant difference between baseline and after 2 h. of Iloprost use. VE/VCO_2_ Single dose of Iloprost: There was a significant difference between baseline and after 30 min of Iloprost use (baseline mean ± SD: 77.0 ± 20.9 vs. after Iloprost mean ± SD: 63.0 ± 14.1; *p* < 0.05). Second dose of Iloprost: There was a significant difference between baseline and after 30 min of Iloprost use (baseline mean ± SD: 77.0 ± 20.9 vs. after Iloprost mean ± SD: 61.7 ± 12.0; *p* < 0.05). After 2 h. of treatment discontinuation: There was no significant difference between baseline and after 2 h. of Iloprost use. Exercise capacity								6MWT Single dose of Iloprost: There was a significant difference between baseline and after 30 min of Iloprost use (baseline mean ± SD: 269 ± 112 m vs. after Iloprost mean ± SD: 324 ± 135 m; *p* < 0.05). Second dose of Iloprost: There was a significant difference between baseline and after 30 min of Iloprost use (baseline mean ± SD: 269 ± 112 m vs. after Iloprost mean ± SD: 330 ± 136 m; *p* < 0.05). After 2 h. of treatment discontinuation: There was no significant difference between baseline and after Iloprost use. Lung function Spirometry FEV_1_ L Single dose of Iloprost: There was no significant difference between baseline and after 30 min of Iloprost use. Second dose of Iloprost: There was no significant difference between baseline and after 30 min of Iloprost use. After 2 h. of treatment discontinuation: There was no significant difference between baseline and after Iloprost use. FVC L Single dose of Iloprost: There was no significant difference between baseline and after 30 min of Iloprost use. Second dose of Iloprost: There was no significant difference between baseline and after 30 min of Iloprost use. After 2 h. of treatment discontinuation: There was no significant difference between baseline and after Iloprost use. Diffusion capacity DLCO Single dose of Iloprost: There was no significant difference between baseline and after 30 min of Iloprost use. Second dose of Iloprost: There was no significant difference between baseline and after 30 min of Iloprost use. After 2 h. of treatment discontinuation: There was no significant difference between baseline and after Iloprost use.

PH, pulmonary hypertension; COPD, Chronic Obstructive Lung Disease; GOLD2, Global Initiative for Chronic Obstructive Lung Disease 2 (moderate COPD); GOLD3, Global Initiative for Chronic Obstructive Lung Disease 2 (severe COPD); PGI_2_, prostaglandin I_2_; mPAP, mean pulmonary artery pressure; RHC, right heart catheterization; ABG, arterial blood gas; WHO-FC, World Health Organization functional class; PaO_2_, partial pressure of oxygen; 6MWT, 6-min walk test; PFT, pulmonary function tests; SGRQ, St George’s respiratory questionnaire; FEV_1_, forced expiratory volume in 1 s; FVC, forced vital capacity; VE, minute ventilation; DLCO, diffusing capacity of the lungs for carbon monoxide; QOL, quality of life; PaCO2, partial pressure of carbon dioxide in arterial blood; SaO_2_, oxygen saturation of arterial blood; D_A–a_ O_2_, alveolar-arterial oxygen gradient; VE/VO_2_, ventilatory equivalent for oxygen; VE/VCO_2_, ventilatory equivalent for carbon dioxide; Qs/Qt, pulmonary shunt fraction; PVR, pulmonary vascular resistance; PAWP, pulmonary arterial wedge pressure; RAP, right atrial pressure; CIx, cardiac index; CO, cardiac output; MAP, mean arterial pressure; SD, standard deviation; N.A, not available.

### 2.1. Data selection

With assistance from a specialist librarian, we searched electronic databases of Medline, Embase, Scopus and Cochrane from inception to 1 February 2023 for publications on the treatment of inhaled prostacyclin in COPD patients (see Appendix S1 for search strategy). Articles describing adult patients with a confirmed diagnosis of COPD-associated pulmonary hypertension who received inhaled drugs targeting the prostacyclin pathway (e.g., iloprost, treprostinil and flolan) were included. We excluded case reports, systematic reviews, review articles, conference abstracts with no full text (since they were not peer-reviewed), non-full-text articles, non-English manuscripts, opinion articles, and book chapters. We did not specify a minimal study sample size for inclusion. To develop focused clinical questions, we used the PICO framework in our search strategy: P: population (patients with a confirmed diagnosis of COPD-associated pulmonary hypertension), I: intervention (inhaled drugs targeting prostacyclin pathway), C: comparison (placebo, usual care), O: outcome (gas exchange, exercise capacity, severity of dyspnoea, lung function and the pulmonary hemodynamics).

### 2.2. Qualitative assessment of study methodology

The assessment of study quality was completed by two authors (AAlq and AAld). We used Cochrane risk-of-bias tools to assess the quality of the studies included in this review (see Appendices S2, S3). For randomised crossover trials, we used the revised Cochrane risk-of-bias tool (31). The tool consists of seven domains: risk of bias arising from the randomisation process, bias arising from period and carryover effects, bias due to deviations from the intended interventions, bias due to deviations from the intended interventions, bias due to missing outcome data, bias in the measurement of the outcome, and bias in the selection of the reported result. Cochrane risk of bias in non-randomised studies assessment was used to assess non-randomised clinical trials (32). The tool consists of seven domains and is similar to that used for randomised crossover trials, except for the first three domains, where non-randomised clinical trial tools focus on bias due to confounding, bias in the selection of participants for the study and bias in classification of interventions. Under each domain, the authors (AAlq and AAld) answered several questions and then classified the risk of bias as low, medium or high. The study was considered to have a low risk of bias if all domains were classified as having a low risk of bias. The study was judged to be at medium risk of bias (non-randomised clinical trial) for or to raise some concerns (for randomised crossover trial) if the domains were marked as low risk and at least one domain was at medium risk of bias.

## 3. Results

**Figure 1:**
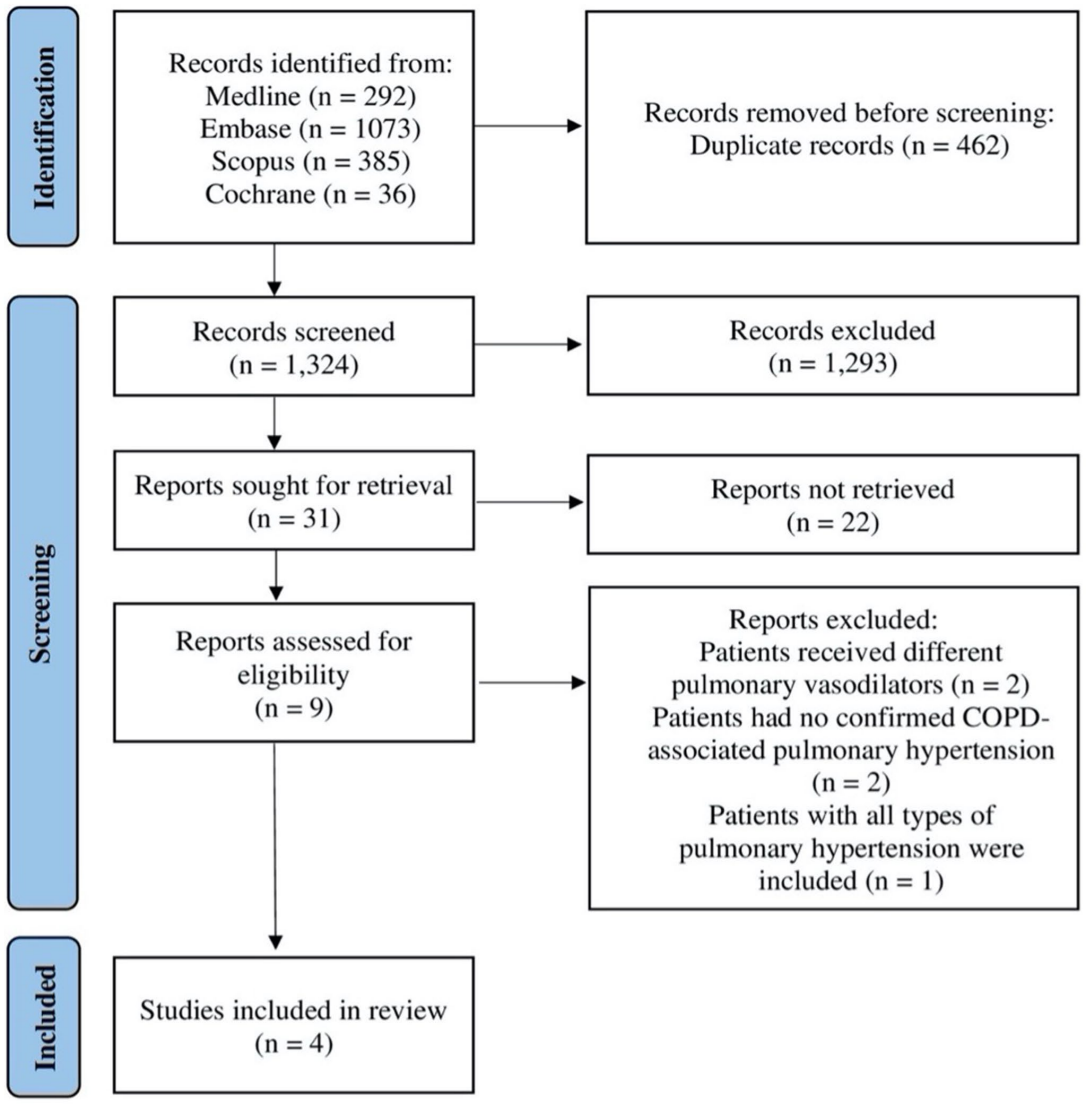
Flow diagram illustrating systematic search and screening strategy based on the Preferred Reporting Items for Systematic Review and Meta-Analysis Guidelines, including numbers of studies meeting eligibility criteria and numbers excluded. COPD: chronic obstructive pulmonary disease.

Initially, the search generated 1,786 studies that were considered potentially eligible. After removing duplicates, 1,324 titles and abstracts were screened. Screening the titles and abstracts resulted in 31 studies assessed according to the inclusion and exclusion criteria. Out of the 31 studies, 22 studies were excluded because they were either conference abstracts or no full texts were available. Thus, nine studies were considered for full-text reading. After reading the full texts of the nine remaining studies, four studies met our inclusion criteria and were included in this systematic review; see Figure 1.

### 3.1. The description of the included studies

The four included studies consisted of a prospective, multicentre, open-label pilot study with a sample size of nine COPD patients conducted in the United States and published in 2017 (27), a prospective, randomised, double-blind, single-centre, crossover trial with a sample size of 16 COPD patients conducted in Switzerland and published in 2012 (28), a prospective, single-centre, open-label trial with a sample size of 67 COPD patients conducted in China and published in 2017 (29), and a prospective, observational study with a sample size of 10 COPD patients conducted in the United States and published in 2010 (30). A full description of the included studies is presented in Table 1.

### 3.2. Gas exchange

Using inhaled prostacyclin analogue treprostinil or iloprost did not significantly affect gas exchange parameters as assessed by the partial pressure of oxygen in arterial blood (PaO_2_), the partial pressure of carbon dioxide in arterial blood (PaCO_2_), the arterial oxygen saturation (SaO_2_), the pulmonary shunt fraction (Qs/Qt), and the alveolar-arterial oxygen concentration gradient (A-a gradient) when compared with baseline measures in two studies (27, 29). In contrast, one study found that using inhaled prostacyclin drug (iloprost) significantly improved all the gas exchange parameters [as assessed by A-a gradient, minute ventilation (VE)/carbon dioxide production ratio (VE/VCO_2_) and VE/oxygen consumption ratio (VE/VO_2_)] compared with baseline measures after 30 min of inhalation of the first and second doses of iloprost (30). However, when these parameters were measured again after 2 h of iloprost use, all gas exchange parameters had returned to baseline level. Taken together, these observations suggest that it is unlikely that inhalation of prostacyclin analogue (treprostinil or iloprost) can improve or worsen gas exchange parameters in patients with pulmonary hypertension due to COPD.

### 3.3. Exercise capacity

Exercise capacity was reported by three studies (27, 28, 30) using 6MWT to assess the effectiveness of inhaled prostacyclin among COPD patients with pulmonary hypertension. Two studies reported no significant effect of inhaled prostacyclin when compared to baseline 6MWT (27) or the placebo group (28). Only one study reported significant improvement in 6MWT compared to baseline among COPD patients with pulmonary hypertension (30). According to a prospective, multicentre, open-label pilot study conducted by Bajwa et al. (27), inhaled treprostinil did not significantly improve 6MWT distance when assessed at week 16 among nine COPD patients with moderate severity and pulmonary hypertension (27). In contrast, in a cohort study conducted by Dernaika et al. (30), a single dose (baseline mean ± SD: 269 ± 112 m vs. after iloprost mean ± SD: 324 ± 135 m; *p* < 0.05) and a second dose (baseline mean ± SD: 269 ± 112 m vs. after iloprost mean ± SD: 330 ± 136 m; *p* < 0.05) of inhaled iloprost significantly improved 6MWT distance compared to baseline when assessed 30 min after treatment among ten severe COPD patients with pulmonary hypertension (30).

### 3.4. Lung function

Two studies used spirometry parameters and diffusion capacity tests as secondary outcomes to assess the effectiveness of inhaled prostacyclin (27, 30). The use of inhaled prostacyclin analogue treprostinil reduced FEV_1_ and forced vital capacity (FVC) but did not affect the diffusion capacity of the lung for carbon monoxide (DLCO) in a prospective, multicentre, open-label pilot study with a sample size of nine COPD patients with pulmonary hypertension (27). In contrast to these findings, improvements in FEV_1_ and FVC (but not DLCO) were reported after 30 min of using the first and second doses of inhaled iloprost compared with baseline measurements (30). Measurements of the same parameters were taken after 2 h of iloprost use. Interestingly, both spirometry parameters and DLCO were not affected compared with the baseline (30). These observations suggest that it is likely that inhaled prostacyclin does not improve lung function in patients with pulmonary hypertension due to COPD.

### 3.5. Severity of dyspnoea

The severity of dyspnoea was measured by two studies (27, 28) using the modified Borg scale to assess the effectiveness of inhaled prostacyclin among COPD patients with pulmonary hypertension. Two studies reported no significant effect of inhaled prostacyclin when compared to the baseline (27) or placebo group (28). According to a prospective, multicentre, open-label pilot study conducted by Bajwa et al. (27), inhaled treprostinil did not significantly improve the severity of dyspnoea when assessed at week 16 among nine COPD patients with moderate severity and pulmonary hypertension (27). Moreover, in a prospective, randomised, double-blind, single-centre, crossover trial study conducted by Boeck et al. (28), inhaled iloprost did not significantly improve the severity of dyspnoea when compared to the placebo group among 16 COPD patients with moderate severity and pulmonary hypertension (28).

### 3.6. The pulmonary hemodynamics

In the studies included in this review, one prospective, single-centre, open-label trial looked at the effect of inhaled iloprost on the pulmonary hemodynamics among patients with COPD-associated pulmonary hypertension (29). For 67 COPD patients with pulmonary hypertension, it was reported that short inhalation of iloprost for 10 min can significantly reduce mean pulmonary artery pressure, pulmonary vascular resistance and pulmonary arterial wedge pressure as compared with baseline. As a result, cardiac output and contractility index were significantly increased in response to inhaled iloprost (29). These findings suggest that inhaled prostacyclin has the potential to improve hemodynamic indices, thereby improving right ventricular function. However, further studies are needed to assess the long-term effect of inhaled prostacyclin on hemodynamic parameters in COPD-associated pulmonary hypertension.

## 4. Discussion

To the best of our knowledge, this is the first systematic review of studies assessing the impact of inhaled prostaglandin I_2_ analogue use on clinical outcomes in patients with pulmonary hypertension due to COPD. Our main findings demonstrated that although inhaled prostacyclin does not seem to improve oxygenation status and COPD-related outcomes (e.g., lung function), inhaled prostacyclin has the potential to reduce mean pulmonary artery pressure and pulmonary vascular resistance, thereby improving right ventricular function in patients with pulmonary hypertension due to COPD. Given that inhaled prostaglandin I_2_ analogue was recently approved for ILD-associated pulmonary hypertension and that the currently available evidence suggests a potential benefit of targeting prostacyclin pathways through the inhaled route, further rigorous randomised clinical trials and observational studies with larger sample sizes are warranted.

It has been reported that the expression of prostaglandin I synthesis (the enzyme responsible for producing prostacyclin) is reduced in the pulmonary arteries of patients with pulmonary hypertension and patients with cigarette smoking-related lung diseases. These observations provide a strong rationale for the use of prostacyclin analogues and prostacyclin receptor agonists in the treatment of these patients. Both oral and inhaled prostacyclin have long been used for group 1 pulmonary hypertension. Despite the high prevalence of COPD-associated pulmonary hypertension, there are currently no approved therapies for these patients due to a lack of evidence. Thus, clinicians have no choice but to use drugs approved for other forms of pulmonary hypertension, particularly group 1. Inhaled prostacyclin, particularly treprostinil for 12 weeks, has been shown to improve exercise capacity in patients with group 1 pulmonary hypertension (33). Recently, a multicentre, randomised, double-blind, placebo-controlled, 16-week trial of 326 patients with pulmonary hypertension due to ILD showed an improvement in exercise capacity (assessed by 6MWT) when using inhaled prostacyclin (34). As a result of this finding, inhaled prostacyclin has recently received United States Food and Drug Administration approval as the first approved drug for group 3 pulmonary hypertension (pulmonary hypertension due to ILD). Given that ILD and COPD share similar clinical presentations and are classified by WHO in one group (1), further studies are needed to find out whether similar effects of inhaled prostacyclin can also be seen in COPD patients.

Interestingly, the findings of two studies included in this systematic review do not support the use of inhaled prostacyclin to improve exercise capacity in COPD patients with pulmonary hypertension when compared to baseline 6MWT (27) or placebo groups (28). Despite the small sample size included in both studies (27, 28) (9 and 16 study populations, respectively) and that one study was stopped due to lower-than-expected enrolment (27), the absence of exercise capacity improvement is likely to be attributable to the fact that 6MWT was stable at baseline in the population of both studies for whom COPD was classified as moderate (GOLD 2) (27, 28). Thus, a further increase in stable 6MWT is less likely. It is interesting to note that when the effect of inhaled prostacyclin on exercise capacity was assessed in patients with severe COPD-associated pulmonary hypertension, an improvement of 6MWT was reported (30), suggesting that inhaled prostacyclin can improve exercise capacity in patients with pulmonary hypertension due to severe (but not moderate) COPD. This plausible speculation is supported by the observation reporting improvement in 6MWT in response to inhaled prostacyclin in the subgroup of patients with COPD who had severe dyspnoea and severe reduction in lung function (28). In support of this, the PERFECT trial which was initiated in 2018 with the aim to mainly evaluate whether inhaled prostacyclin can improve exercise capacity in those with pulmonary hypertension due to COPD has recently been terminated following a routine safety and efficacy analysis conducted by the data safety monitoring committee (35). Although the findings of the PERFECT trial are not yet published and the available evidence to date points against the use of inhaled prostacyclin to improve exercise capacity, there is still an unmet need for an appropriately powered multicentre, randomised, double-blind, placebo-controlled crossover trial of inhaled prostacyclin impact on exercise capacity and other clinical outcomes with COPD and pulmonary hypertension severity stratification. This together with, the currently ongoing clinical trial conducted to assess the effect of inhaled soluble guanylate cyclase stimulator on exercise capacity (36), can provide clear evidence on the use of selective inhaled pulmonary vasodilators for COPD-associated pulmonary hypertension.

Lung function, particularly spirometry parameters (e.g., FEV_1_ and FVC) are helpful tests used to diagnose, follow, and manage patients with COPD. In addition to its importance in the diagnosis of COPD, DLCO is a known predictor for survival in patients with pulmonary hypertension (37). The fact that the findings of the studies included in the review demonstrated no improvement of lung function (27, 30) or the severity of dyspnoea (27, 28) suggests that inhaled prostacyclin is unlikely to improve COPD-related outcomes in COPD patients with pulmonary hypertension. Unlike the findings of this systematic review, a post-hoc analysis of the INCREASE study demonstrated an improvement of FVC as compared with placebo at 16 weeks in patients with pulmonary hypertension due to ILD (38). The ways in which COPD is different than ILD in terms of clinical phenotype, treatment response and outcomes and the fact that treprostinil has antifibrotic effects (likely though the activation of the prostaglandin E receptor 2) (39) could explain why the improvement of FVC was only seen in those with pulmonary hypertension due to ILD but not COPD. This is supported by the observation of INCREASE study demonstrating most improvement of FVC in patients with idiopathic pulmonary fibrosis (IPF) (38) which paved the way for the ongoing TETON trial that aimed to study the effect of inhaled treprostinil on FVC in IPF patients without pulmonary hypertension (39).

Pulmonary hypertension is defined as increased mean pulmonary artery pressure and pulmonary vascular resistance. Routine assessment of these hemodynamic parameters is needed to monitor the effectiveness of pulmonary hypertension drugs. However, since non-invasive measurement of the pulmonary hemodynamics is inaccurate in those with COPD due to lung hyperinflation (8, 40, 41) and assessment using right-heart catheterisation is considered to be invasive and time-consuming procedure, only one study to date has assessed the effect of inhaled prostacyclin (iloprost) on hemodynamic values in COPD patients with pulmonary hypertension (29). The substantial improvement in mean pulmonary artery pressure, pulmonary vascular resistance and cardiac output in response to the inhalation of prostacyclin (29) supports the use of inhaled prostacyclin analogue in the treatment of patients with COPD-associated pulmonary hypertension. However, it should be noted that the short-term design of this study restricts the findings to only short-term use. This indicates that urgent clinical trials are needed to assess the long-term effect of inhaled prostacyclin in COPD-associated pulmonary hypertension. The findings of a study conducted by Wang et al. are supported by other studies that showed an improvement of hemodynamic parameters in COPD patients with pulmonary hypertension after the use of sildenafil (enhances nitric oxide *via* the inhibition of phosphodiesterase type 5) (42) and inhaled nitric oxide (43). Collectively, these findings suggest that selective pulmonary vasodilators approved for the treatment of group 1 pulmonary hypertension can be used to improve hemodynamic parameters in COPD patients with pulmonary hypertension.

Despite the improvement of hemodynamic parameters, the use of systemically administered pulmonary vasodilators was associated with a deleterious effect on gas exchange. In patients with COPD, it is thought that systemically administered pulmonary vasodilators (e.g., sildenafil) can dilate vasculature around both ventilated and non-ventilated, leading to inhibition of hypoxic pulmonary vasoconstriction and impairment of gas exchange (42, 44). When pulmonary vasodilators, including prostacyclin, are used through the inhalation route in patients with group 3 pulmonary hypertension, the risk of ventilation and perfusion mismatch associated with systemic vasodilators use can be minimised (45), considering that inhaled pulmonary vasodilator use in these patients can target the better-ventilated alveoli. In this review, the findings of two studies demonstrating that inhaled prostacyclin did not affect the gas exchange index (e.g., A-a gradient) when compared with baseline measures (27, 29), suggesting that using the selective pulmonary vasodilator (inhaled) route is less likely to cause ventilation and perfusion mismatch in COPD patients with pulmonary hypertension. This is further supported by the observation showing the improvement of several gas exchange parameters in patients with COPD-associated pulmonary hypertension 30 min after the use of inhaled prostacyclin (30). It is interesting to note that these parameters returned to baseline 2 h after stable prostacyclin analogue inhalation. This is likely due to the short-term effects of prostacyclin leveling off within 30–60 min (46), which requires repetitive administration. Despite this limitation, the current evidence demonstrates the superiority of inhaled vasodilators (e.g., inhaled prostacyclin) over oral vasodilators (e.g., sildenafil) for the treatment of patients with COPD-associated pulmonary hypertension as inhaled vasodilators can divert blood to better-ventilated alveoli, thereby minimising the mismatched distribution of ventilation and perfusion.

### 4.1. Strength and limitation

To the best of our knowledge, this is the first systematic review to summarise the current evidence to assess the impact of inhaled prostaglandin I_2_ analogue use on the pulmonary hemodynamics, exercise function, lung function, and oxygenation status in patients with pulmonary hypertension due to COPD. We included both randomised trials and observational studies. However, our study had some limitations. Studies included in this review were of a short duration of follow-up. Moreover, our results should be interpreted with caution because of the small sample size in the studies included in this review.

## 5. Conclusion

The findings of the systematic review suggest that the use of inhaled prostacyclin has the potential to improve hemodynamic parameters in patients with COPD-associated pulmonary hypertension without impairing gas exchange, but conclusive benefits were not demonstrated for other clinical outcomes (e.g., lung function and exercise capacity). There is an unprecedented unmet need for a large randomised controlled trial to further evaluate the potential benefit of inhaled prostacyclin analogue for the treatment of pulmonary hypertension due to COPD.

## Data availability statement

The original contributions presented in the study are included in the article/supplementary material, further inquiries can be directed to the corresponding author.

## Author contributions

AAlq, HB, and AAld contributed to the conception and design of the review. AAlq, HB, AAld, HA, RS, and MM contributed to data extraction. AAlq, AAld, JA, AAlG, AN, SA, and HA interpret data, and wrote sections of the manuscript. All authors contributed to the article and approved the submitted version.

## Conflict of interest

The authors declare that the research was conducted in the absence of any commercial or financial relationships that could be construed as a potential conflict of interest.

## Publisher’s note

All claims expressed in this article are solely those of the authors and do not necessarily represent those of their affiliated organizations, or those of the publisher, the editors and the reviewers. Any product that may be evaluated in this article, or claim that may be made by its manufacturer, is not guaranteed or endorsed by the publisher.

## Supplementary material

The Supplementary material for this article can be found online at: https://www.frontiersin.org/articles/10.3389/fmed.2023.1217156/full#supplementary-material

## Footnotes

^1^ https://www.rayyan.ai/
