# Supplementary material for: Inhaled therapies targeting prostacyclin pathway in pulmonary hypertension due to COPD: systematic review
Source: Front Med (Lausanne). 2023 Aug 29;10:1217156. doi: 10.3389/fmed.2023.1217156 (PMC10496018; doi:10.3389/fmed.2023.1217156)
Supplement: Supplementary file 1 [file Data_Sheet_1.docx]

Supplementary Material

Inhaled therapies targeting prostacyclin pathway in pulmonary hypertension due to COPD: systematic review

**Abdullah A. Alqarni^1,2^*, Abdulelah M. Aldhahir^3^, Heba M. Bintalib^4,5^, Jaber S. Alqahtani^6^, Rayan A. Siraj^7^, Mansour Majrshi^8,9^, Abdulkareem A. AlGarni^10,11^, Abdallah Y. Naser^12^, Sara A. Alghamdi^13^ and Hassan Alwafi^14^


^1^Department of Respiratory Therapy, Faculty of Medical Rehabilitation Sciences, King Abdulaziz University, Jeddah, Saudi Arabia, ^2^Respiratory Therapy Unit, King Abdulaziz University Hospital, Jeddah, Saudi Arabia, ^3^Respiratory Therapy Department, Faculty of Applied Medical Sciences, Jazan University, Jazan, Saudi Arabia, ^4^Department of Respiratory Care, King Saud bin Abdulaziz University for Health Sciences, Jeddah, Saudi Arabia, ^5^King Abdullah International Medical Research Centre, Jeddah, Saudi Arabia, ^6^Department of Respiratory Care, Prince Sultan Military College of Health Sciences, Dammam, Saudi Arabia, ^7^Department of Respiratory Care, College of Applied Medical Sciences, King Faisal University, Al Ahsa, Saudi Arabia, ^8^National Heart and Lung Institute, Imperial College London, London, UK, ^9^Respiratory Medicine, Royal Brompton Hospital, London, UK, ^10^King Abdulaziz Hospital, The Ministry of National Guard Health Affairs, Al Ahsa, Saudi Arabia, ^11^King Saud bin Abdulaziz University for Health Sciences, College of Applied Medical Sciences, Al Ahsa, Saudi Arabia, ^12^Department of Applied Pharmaceutical Sciences and Clinical Pharmacy, Faculty of Pharmacy, Isra University, Amman, Jordan, ^13^Respiratory Care Department, Mediclinic Almurjan Hospital, Jeddah, Saudi Arabia, ^14^Faculty of Medicine, Umm Al-Qura University, Mecca, Saudi Arabia**

*** Correspondence:**Abdullah A. Alqarni; aaalqarni1@kau.edu.sa

**Appendices**

Search strategy for the systematic review

# Table S1: Search strategy in Medline, Embase, Cochrane and Scopus

| **Search strategy in Ovid MEDLINE(R) and embase** |
| --- |
|  |
| 1 exp Lung Diseases, Obstructive/ 231884 |
| 2 (chronic adj2 (air* adj2 obstruct*)).kf,fx,tw. 2305 |
| 3 ((lung* or pulmon* or respirat* or bronchopulmon*) adj3 obstruct*).kf,fx,tw. 73558 |
| 4 (COAD or COBD or COPD).kf,fx,tw. 56809 |
| 5 ((centriacinar* or centrilobular* or focal or panacinar* or panlobular* or pulmonar*) adj2 emphysem*).kf,fx,tw. 7378 |
| 6 exp Bronchitis/ 31090 |
| 7 "bronchit*".ab,kf,ti,fx. 24800 |
| 8 exp Hypoxia/ 87544 |
| 9 hypoxia.kf,fx,tw. 132808 |
| 10 (oxygen adj4 (lack* or deprivation*)).kf,fx,tw. 9261 |
| 11 1 or 2 or 3 or 4 or 5 or 6 or 7 278312 |
| 12 8 or 9 or 10 179321 |
| 13 11 or 12 453757 |
| 14 exp Hypertension/ 311077 |
| 15 (hypertension or (blood adj2 pressure*)).kf,fx,tw. 673464 |
| 16 14 or 15 738087 |
| 17 exp Epoprostenol/ 12873 |
| 18 (Epoprostenol or prostacyclin or treprostinil or iEPO or PGI2 or tyvaso or flolan).kf,fx,tw. 18577 |
| 19 exp Iloprost/ 2110 |
| 20 (iloprost or ventavis).kf,fx,tw. 2625 |
| 21 17 or 18 or 19 or 20 22680 |
| 22 13 and 16 24465 |
| 23 21 and 22 333 |
| 24 Limit 23 to English |
| **Search strategy in Scopus** |
| ( TITLE-ABS-KEY ( *obstructive AND lung AND diseases* ) OR TITLE-ABS-KEY ( ( chronic AND air* AND obstruct* ) ) OR TITLE-ABS-KEY ( ( coad OR cobd OR copd ) ) OR TITLE-ABS-KEY ( ( ( centriacinar* OR centrilobular* OR focal OR panacinar* OR panlobular* OR pulmonar* ) emphysem* ) ) OR TITLE-ABS-KEY ( bronchitis ) OR TITLE-ABS-KEY ( ( oxygen AND lack* OR deprivation* ) ) AND TITLE-ABS-KEY ( hypertension ) OR TITLE-ABS-KEY ( ( hypertension OR ( *blood AND pressure* ) ) ) AND TITLE-ABS-KEY ( ( epoprostenol OR prostacyclin OR treprostinil OR iepo OR pgi2 OR tyvaso OR flolan ) ) OR TITLE-ABS-KEY ( iloprost OR ventavis ) ) AND ( LIMIT-TO ( LANGUAGE , "English" ) ) |
| **Search strategy in Cochrane** |
| ID Search Hits |
| #1 MeSH descriptor: [Lung Diseases, Obstructive] explode all trees 20954 |
| #2 (chronic NEXT (air* and obstruct*)):ti,ab,kw OR ((coad OR cobd OR copd)):ti,ab,kw OR ((centriacinar* OR centrilobular* OR focal OR panacinar* OR panlobular* OR pulmonar* ) NEXT (emphysem* )):ti,ab,kw OR ((lung* or pulmon* or respirat* or bronchopulmon*) NEXT (obstruct*)):ti,ab,kw (Word variations have been searched) 23974 |
| #3 MeSH descriptor: [Bronchitis] explode all trees 1907 |
| #4 (bronchit*):ti,ab,kw (Word variations have been searched) 4540 |
| #5 #1 or #2 or #3 or #4 39651 |
| #6 MeSH descriptor: [Hypoxia] explode all trees 2433 |
| #7 (hypoxia):ti,ab,kw 7295 |
| #8 (oxygen NEXT (lack* or deprivation*)):ti,ab,kw 23 |
| #9 #6 or #7 or #8 7310 |
| #10 #5 or #9 46568 |
| #11 MeSH descriptor: [Hypertension] explode all trees 20110 |
| #12 (hypertension or (blood NEXT pressure*)):ti,ab,kw 139798 |
| #13 #11 or #12 139798 |
| #14 MeSH descriptor: [Epoprostenol] explode all trees 542 |
| #15 ((Epoprostenol or prostacyclin or treprostinil or iEPO or PGI2 or tyvaso or flolan)):ti,ab,kw 1667 |
| #16 MeSH descriptor: [Iloprost] explode all trees 228 |
| #17 ((iloprost or ventavis)):ti,ab,kw 508 |
| #18 #14 or #15 or #16 or #17 1965 |
| #19 #10 AND #13 3827 |
| #20 #18 AND #19 36 |

Risk Of Bias Assessment

**Table S2: Summary of the Cochrane risk of bias in non-randomized studies assessment**

| Domain | Bajawa et al, 2017 | Wang et al. 2017 | Dernaika et al. 2010 |
| --- | --- | --- | --- |
| Bias due to confounding | Low | Low | Low |
| Bias in selection of participants into the study | Low | Low | Low |
| Bias in classification of interventions | Low | Low | Low |
| Bias due to deviations from intended interventions | Low | Low | Low |
| Bias due to missing data | Low | Low | Low |
| Bias in measurement of outcomes | Low | Low | Low |
| Bias in selection of the reported result | Medium | Low | Low |
| Overall bias | Medium bias | Low bias | Low bias |

**Table S3: Summary of the Cochrane risk of bias in randomized studies (crossover) trials assessment**

| Domain | Boeck et al. 2012 |
| --- | --- |
| Risk of bias arising from the randomization process | Low |
| Risk of bias arising from period and carryover effects | Low |
| Risk of bias due to deviations from the intended interventions (effect of assignment to intervention) | Low |
| Risk of bias due to deviations from the intended interventions (effect of adhering to intervention) | Low |
| Risk of bias due to missing outcome data | Low |
| Risk of bias in measurement of the outcome | Low |
| Risk of bias in selection of the reported result | Low |
| Overall bias | Low bias |
